# Rx3 and Shh direct anisotropic growth and specification in the zebrafish tuberal/anterior hypothalamus

**DOI:** 10.1242/dev.138305

**Published:** 2016-07-15

**Authors:** Victor Muthu, Helen Eachus, Pam Ellis, Sarah Brown, Marysia Placzek

**Affiliations:** 1 The Bateson Centre andDepartment of Biomedical Science, University of Sheffield, Sheffield S10 2TN, UK; 2 Department of Genetics, University of Pennsylvania, Philadelphia, PA 19104, USA

**Keywords:** Hypothalamus development, Anterior hypothalamus, Rx3, Sonic hedgehog, Tuberal hypothalamus, Zebrafish hypothalamus

## Abstract

In the developing brain, growth and differentiation are intimately linked. Here, we show that in the zebrafish embryo, the homeodomain transcription factor Rx3 coordinates these processes to build the tuberal/anterior hypothalamus. Analysis of *rx3 chk* mutant/*rx3* morphant fish and EdU pulse-chase studies reveal that *rx3* is required to select tuberal/anterior hypothalamic progenitors and to orchestrate their anisotropic growth. In the absence of Rx3 function, progenitors accumulate in the third ventricular wall, die or are inappropriately specified, the *shh^+^* anterior recess does not form, and its resident *pomc^+^*, *ff1b^+^* and *otpb^+^* Th1^+^ cells fail to differentiate. Manipulation of Shh signalling shows that Shh coordinates progenitor cell selection and behaviour by acting as an on-off switch for *rx3*. Together, our studies show that Shh and Rx3 govern formation of a distinct progenitor domain that elaborates patterning through its anisotropic growth and differentiation.

## Abstract (precis)

**Summary:** During zebrafish tuberal/anterior hypothalamus development, Shh and Rx3 govern the formation of a distinct progenitor domain that elaborates pattern through its anisotropic growth and differentiation.

## INTRODUCTION

The hypothalamus is an ancient part of the ventral forebrain. It centrally regulates homeostatic processes that are essential to survival and species propagation, including autonomic regulation of energy balance, growth, stress and reproduction. Such adaptive functions are dependent upon the integrated function of evolutionarily conserved neurons (reviewed by Bedont et al., 2015; Biran et al., 2015; Burbridge et al., 2016; Löhr and Hammerschmidt, 2011; Machluf et al., 2011; Pearson and Placzek, 2013; Puelles et al., 2012) that, in mouse, are located within defined nuclei, including the arcuate nucleus (Arc) and ventromedial nucleus (VMN) of the tuberal hypothalamus, and the paraventricular nucleus (PVN) of the anterior hypothalamus. In zebrafish, functionally analogous neurons exist in the periventricular tuberal (pevTub) hypothalamus and the neurosecretory preoptic (NPO) area (Biran et al., 2015; Herget et al., 2014; see Materials and Methods and Discussion for terminology). Many transcription factors and signalling ligands that govern differentiation of hypothalamic neurons from progenitor cells have also been largely conserved (reviewed by Bedont et al., 2015; Biran et al., 2015; Burbridge et al., 2016; Pearson and Placzek, 2013; Puelles et al., 2012).

The mechanisms through which secreted signalling ligands and transcription factors define and build hypothalamic territories and cells remain enigmatic (see Bedont et al., 2015; Puelles et al., 2012; Pearson and Placzek, 2013). Models based on the uniform growth and differentiation of patterned territories do not account for the complex spatial patterns of the hypothalamus or the protracted period of hypothalamic neuronal differentiation and, at present, little is known about how early patterning events are elaborated over time. In the hypothalamus, distinct neural progenitor domains that form around the third (diencephalic) ventricle (3V) are not as well-characterized as those in other regions of the CNS. Moreover, the third ventricle is sculpted into the infundibular, optic, and other smaller and ill-defined recesses in mammals (Amat et al., 1992; O'Rahilly and Muller, 1990), and lateral (LR), posterior (PR) and anterior (AR) recesses in zebrafish (Wang et al., 2009, 2012). Three unexplored questions are when such hypothalamic recesses form, whether they are composed of distinct progenitor cells and whether their appearance correlates with the emergence of particular neuronal subsets.

The paired-like homeodomain transcription factor *Rax* (also known as *Rx*) and its fish orthologue, *rx3*, are expressed within retinal and hypothalamic progenitors (Bailey et al., 2004; Bielen and Houart, 2012; Cavodeassi et al., 2013; Chuang et al., 1999; Furukawa et al., 1997; Lu et al., 2013; Mathers et al., 1997; Stigloher et al., 2006; Medina-Martinez et al., 2009; Muranishi et al., 2012; Pak et al., 2014; Zhang et al., 2000) and play a central role in eye development. Disruption of *Rx* leads to small or absent eyes in mouse (Bailey et al., 2004; Mathers et al., 1997; Medina-Martinez et al., 2009; Muranishi et al., 2012; Zhang et.al., 2000) and is associated with anophthalmia in humans (Voronina et al., 2004). In zebrafish, loss of function of Rx3, including mutation in the zebrafish *rx3* gene (*chk* mutant), disrupts eye morphogenesis (Kennedy et al., 2004; Loosli et al., 2003; Stigloher et al., 2006): retinal progenitors are specified, but remain trapped in the lateral wall of the diencephalon, failing to undergo appropriate migration (Rembold et al., 2006) and differentiation (Stigloher et al., 2006).

In addition to its well-documented role in eye formation, *Rx/rx3* governs hypothalamic development. *Rx*-null mice show variable penetrance, but all display abnormalities in the ventral hypothalamus (Mathers et al., 1997; Medina-Martinez et al., 2009; Zhang et al., 2000). Lineage-tracing studies demonstrate that *Rx^+^* progenitors give rise to *Sf1 *(*Nr5a1*)*^+^* VMN and *Pomc^+^* Arc tuberal neurons, and targeted ablation of *Rx* in a subset of VMN progenitors leads to a fate switch from an *Sf1^+^* VMN identity to a *Dlx2^+^* dorsomedial nucleus (DMN) identity (Lu et al., 2013). These studies suggest that *Rx* functions in progenitor cells to cell-autonomously select *Sf1^+^* VMN and *Pomc^+^* Arc identities. In zebrafish, *chk* mutants and *rx3* morphants similarly show reduced numbers of pevTub *pomc^+^* neurons and additionally decreased NPO *avp^+^* (formerly *vt*, arginine vasotocin) neurons (Dickmeis et al., 2007; Tessmar-Raible et al., 2007), although currently the underlying mechanism is unclear. These studies, together, raise the possibility that *Rx/rx3* plays a widespread role in the differentiation of tuberal and anterior/NPO hypothalamic neurons.

In mice, expression of the secreted signalling ligand *Shh* overlaps with that of *Rx* (Shimogori et al., 2010) and conditional ablation of *Shh* from the anterior-basal hypothalamus results in phenotypes that resemble the loss of *Rx*, including a reduction/loss of *Avp^+^* PVN and *Pomc^+^* Arc neurons (Shimogori et al., 2010; Szabo et al., 2009). As yet, however, the link between Shh and Rx/Rx3 remains unclear and the mechanisms that operate downstream of Shh and Rx/Rx3 to govern hypothalamic differentiation are unresolved.

Here, we analyse *rx3* and *shh* expression and function in the developing zebrafish hypothalamus. Analysis of *chk* mutant and *rx3* morphant fish, together with 5-ethynyl-2′-deoxyuridine (EdU) pulse-chase experiments, show that Rx3 is required for a switch in progenitor domain identity, and for the survival and anisotropic growth of tuberal/anterior progenitors, including their progression to *rx3^−^shh^+^* AR cells and to *pomc^+^*, *ff1b* (*nr5a1a*)*^+^* and *otpb^+^* Th1 (Th)^+^ tuberal/anterior fates. Timed delivery of cyclopamine or SAG reveals that Shh signalling governs these processes via dual control of *rx3* expression, inducing then downregulating it. We demonstrate that *rx3* downregulation, mediated by Shh signalling, is an essential component of Rx3 function: failure to downregulate *rx3* leads to the failure of anisotropic growth, loss of the *shh*^+^*rx3*^−^ AR and failure of tuberal/anterior cell differentiation. Together, our studies reveal a mechanism that elaborates early patterning around the hypothalamic ventricle by the selective growth of distinct progenitor cells.

## RESULTS

### *rx3* expression in third ventricle cells

**Fig. 1.**
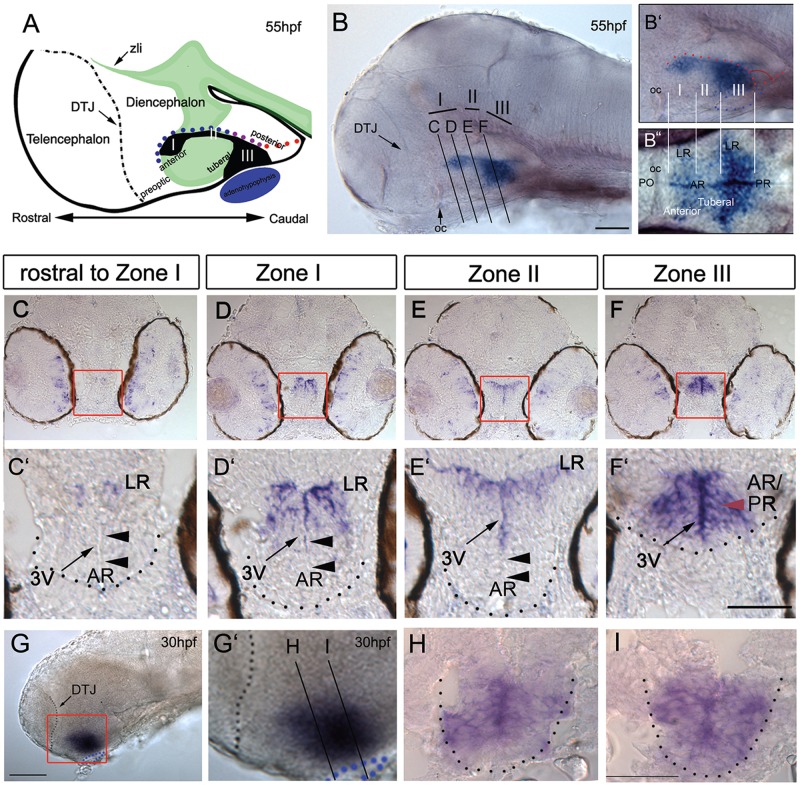
***rx3* expression around the third ventricle.** (A) Schematic of 55 hpf forebrain indicating subdivisions of hypothalamus relative to the rostro-caudal axis and adenohypophysis (blue oval). Green and black show *shh* (Fig. 3) and *rx3* expression. Dots depict rostro-caudal position of AR (blue) and PR (red) next to zone III (purple). (B-B″) Whole-mount 55 hpf embryo after *rx3 in situ* hybridization. In B, lines show planes of section shown in C-F. In B′,B″ side and ventral views are aligned (white lines) and show position of *rx3* relative to morphological landmarks (oc, optic commissure; PO, preoptic hypothalamus). (C-F′) Representative serial sections through a single embryo: bottom panels show high-power views of boxed regions. Red arrowheads point to zone III neuroepithelial-like cells; black arrowheads point to *rx3^−^* cells in AR tips. (G-I) Whole-mount side view of 30 hpf embryo after *rx3 in situ* hybridization; lines in G′ show planes of sections shown in H,I. Dotted lines in C′-F′,H,I delineate outline of ventral hypothalamus, and in G,G′ delineate DTJ. zli, zona limitans intrathalamica. Scale bars: 50 µm.

Previous studies have described zebrafish *rx3* expression (Bielen and Houart, 2012; Cavodeassi et al., 2013; Chuang et al., 1999; Kennedy et al., 2004; Loosli et al., 2003; Stigloher et al., 2006) but have not performed a detailed analysis in the 2- to 3-day embryo. Neurons in the hypothalamus, including *pomc^+^* and *avp^+^* neurons that are decreased/lost in the absence of *rx3* (Dickmeis et al., 2007; Tessmar-Raible et al., 2007) begin to differentiate over the first 2-3 days of development (Liu et al., 2003; Dickmeis et al., 2007; Tessmar-Raible et al., 2007) and we therefore focused on this period. At 55 hours post-fertilization (hpf), *rx3* is detected in three adjacent zones in the hypothalamus (Fig. 1A-B″). In keeping with mouse nomenclature (Lu et al., 2013), we term these zones I, II and III, characterized by the thin strip of weakly *rx3*-positive [*rx3^(weak+)^*] cells in zone II. Sections show that at its rostral limit, in zone I, *rx3* is expressed in neuroepithelial-like cells around the AR and LR of the third ventricle (Fig. 1C,D) but is excluded from the AR tips (Fig. 1C′D′, arrowheads). In zone II, *rx3* labels cells that closely line the AR/LR, again excluded from the AR tips (Fig. 1E,E′, arrowheads). In zone III, *rx3* marks neuroepithelial-like cells around the third ventricle, which in this region (between anterior and posterior recesses, see Fig. 1A,B″) is small (Fig. 1F,F′). At 30 hpf, the entire third ventricle is small and lined throughout by *rx3*^+^ neuroepithelial-like cells (Fig. 1G-I). Thus, the well-defined recesses of the third ventricle, and characteristic *rx3^+^* profiles, develop over 30-55 hpf.

### Tuberal/anterior hypothalamus elongates from proliferating *rx3^+^* progenitors

**Fig. 2.**
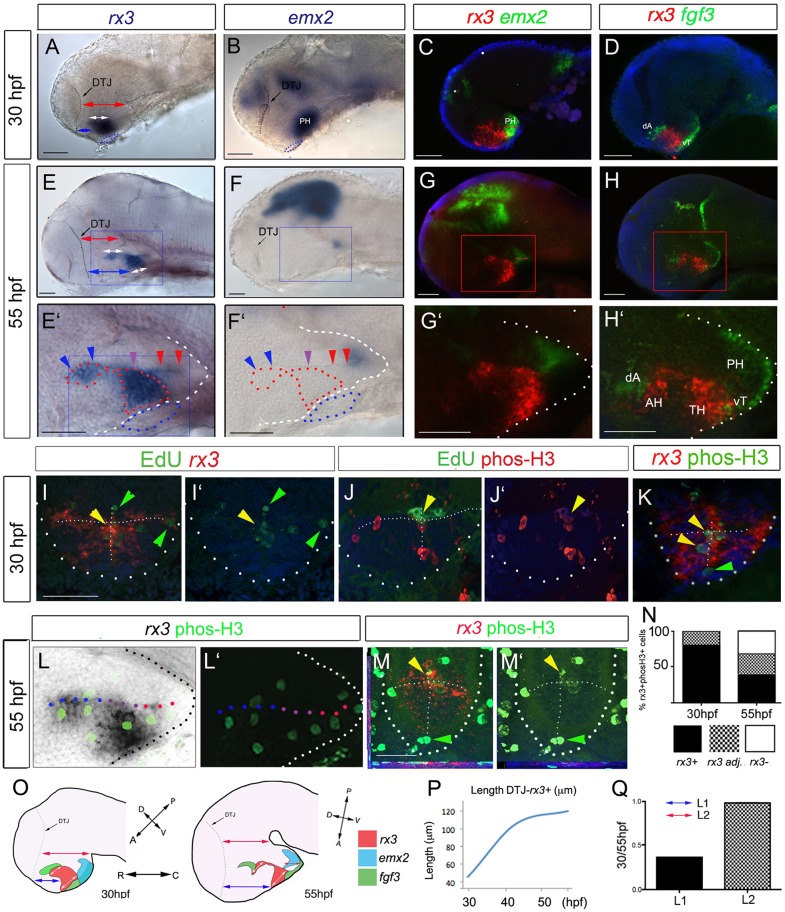
**Anterior/tuberal hypothalamus elongates from *rx3^+^* progenitors.** (A-H′) Side views after single or double FISH at 30 hpf (A-D) and 55 hpf (E-H; E′-H′ show high-power views of boxed regions). Arrows in A,C,E,G show distances measured for growth comparisons. Arrowheads in E′,F′ indicate position of recesses (colour-coded as in Fig. 1A). (I-K) Maximum intensity projections of representative sections through 30 hpf embryos. I,J show serial adjacent sections; I′,J′ show single-channel views. Arrowheads show co-labelled (yellow) or single-labelled (green) cells. T-shaped white dotted lines indicate outline of AR and LR. (L,L′) Side views of 55 hpf embryo; L′ shows single-channel view. (M,M′) Representative single-plane views taken through zone II; M′ shows single-channel view. Yellow arrowheads show double-labelled cells; green arrowheads point to phosH3^+^ *rx3^−^* cells at recess tips. (N) Quantitative analyses of cycling cells at 30-55 hpf as indicated by phosH3 expression in *rx3*^+^ cells, *rx3*^−^ cells or in cells adjacent (adj.) to *rx3*^+^ cells. (O) Schematic depicting *rx3*, *fgf3* and *emx2* expression, and change in length and axial orientation of hypothalamus. A ‘bending’ of the tuberal/anterior hypothalamus occurs over 30-55 hpf, relative to the rostro-caudal axis. Red arrows indicate length of dorsal diencephalon or length of *emx2^+^* PH; white arrows indicate length of *rx3*^+^ territories; blue arrows indicate distance from DTJ to *rx3^+^* zone III. (P) Length from DTJ to rostral tip of *rx3^+^* zone III (*n*=5 embryos each at 30, 40, 48, 55 hpf). (Q) Tuberal/anterior hypothalamus grows approx. 2.5-fold more than dorsal diencephalon, *emx2^+^* PH or ventral *rx3^+^* zone III (*n*=10 each; *P*<0.0001). Dotted and dashed lines delineate ventral hypothalamus and T-shaped AR/LR (white), adenohypophysis (blue) and *rx3*-expressing domain (red). AH, anterior hypothalamus; dA, dorso-anterior; PH, posterior hypothalamus; TH; tuberal hypothalamus; vT, ventral tuberal. Scale bars: 50 µm.

To determine the position of *rx3^+^* cells relative to other hypothalamic regions, we compared *rx3* expression with that of *emx2* and *fgf3*, which mark the posterior, ventro-tuberal and dorso-anterior hypothalamus (Herzog et al., 2004; Kapsimali et al., 2004; Liu et al., 2013; Mathieu et al., 2002), and with the position of the adenohypophysis and the diencephalic-telencephalic junction (DTJ), which are morphologically distinct landmarks. Over 30-55 hpf, *rx3* expression is rostral and largely complementary to *emx2*, and is sandwiched between ventro-tuberal and dorso-anterior *fgf3^+^* cells (Fig. 2A-H′, schematics in 2O), and in zone III it overlies the adenohypophysis. This suggests that throughout 30-55 hpf *rx3* demarcates cells at the boundary of the posterior and tuberal/anterior hypothalamus.

Prior to 30 hpf, *rx3* is expressed in progenitor cells (Bielen and Houart, 2012; Cavodeassi et al., 2013; Loosli et al., 2003; Rembold et al., 2006; Stigloher et al., 2006) and the third ventricle is known to harbour cycling cells (Bosco et al., 2013; Lee et al., 2006; Wang et al., 2009, 2012; Wullimann et al., 1999). To address directly whether 30 hpf *rx3^+^* cells proliferate, we pulsed fish with EdU, culled immediately, and analysed sections for EdU and *rx3* expression (Fig. 2I). At 30 hpf, 77% EdU^+^ cells are *rx3^+^* and the remainder immediately abut *rx3^+^* cells (Fig. 2I,I′; *n*=110 cells, 4 embryos). Co-analysis of alternate sections with EdU and phosphorylated histone H3 antibody (phosH3) shows that cells in S phase progress to M phase (Fig. 2J,J′). Analysis of control embryos with phosH3 and *rx3* confirms that the majority of cycling cells at 30 hpf are *rx3^+^* (68% phosH3^+^ cells co-express *rx3*; 32% phosH3^+^ cells abut *rx3^+^* cells; Fig. 2K,N; *n*=76 cells, 4 embryos). Whole-mount views of embryos double-labelled with *rx3* and phosH3 suggests that by 55 hpf, fewer cycling cells are *rx3^+^* (Fig. 2L,L′). Sections confirm this, showing that at 55 hpf 35% cycling cells are *rx3^+^*, 28% abut *rx3^+^* cells but 38% are now detected in the *rx3^−^* recess tips (Fig. 2M,N; *n*=92 cells, 4 embryos).

Although expressed in proliferating cells, the rostro-caudal length of *rx3* expression in zones I and III does not change over 30-55 hpf (Fig. 2A,E,O,P) indicating its dynamic regulation. Proliferation correlates, though, with rostro-caudal growth of the tuberal/anterior hypothalamus (Fig. 2A,E,O,P). Growth is greatest over 30-48 hpf (Fig. 2P), and is 2.5-fold greater than rostro-caudal growth of the posterior hypothalamus or the dorsal diencephalon over this period (Fig. 2Q). In summary, the tuberal/anterior hypothalamus shows anisotropic growth over 30-55 hpf, driven from proliferating *rx3^+^* cells and their immediate neighbours.

### Development of *rx3^−^shh^+^* AR and tuberal/anterior immature neurons

**Fig. 3.**
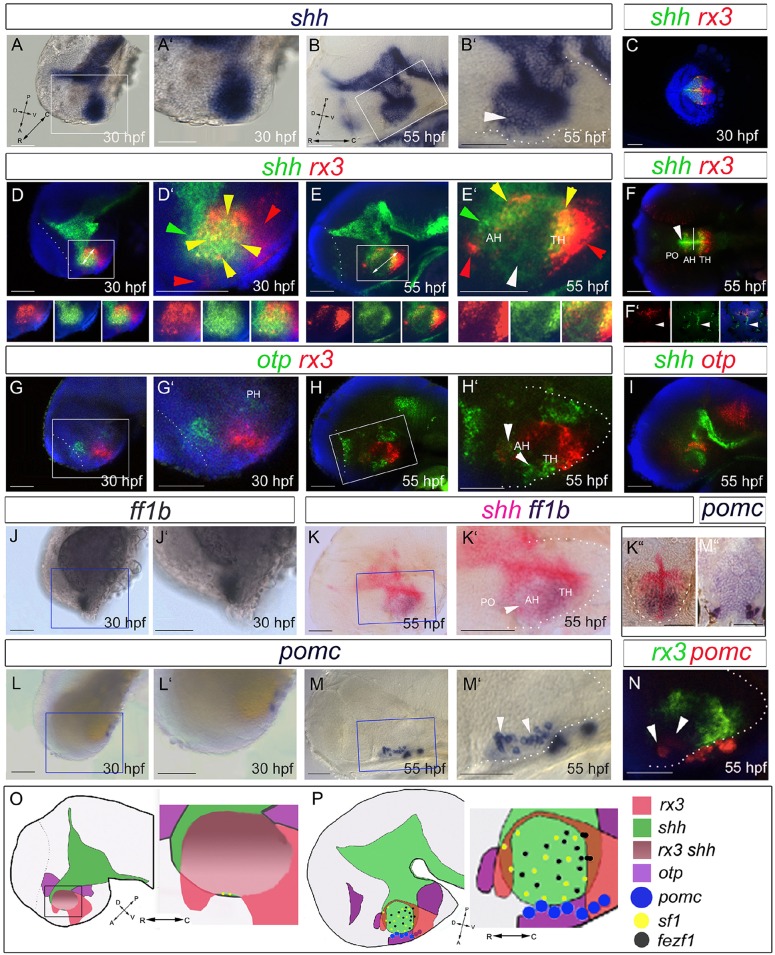
**Differentiation in the 30-55 hpf anterior/tuberal hypothalamus.** (A-N) Side views (A,B,D,E,G-J,L-N), ventral views (C,F), sagittal (K) or transverse (K″,M″) sections of 30 hpf and 55 hpf embryos. A′,M′ show high-power views of boxed regions. In B′,E′,F, white arrowheads point to *shh^(weak+)^* AR cells; in H′, to *otpb^+^* cells in the tuberal/anterior hypothalamus; in M′,N, to hypothalamic *pomc^+^* cells. In D′,E′, arrowheads point to *rx3^+^shh^+^* cells (yellow), *rx3^+^* cells (red) or *shh^+^* cells (green). (O,P) Schematics depicting expression domains at 30 hpf (O) or 55 hpf (P). AH, anterior hypothalamus; PH, posterior hypothalamus; PO, preoptic hypothalamus; TH; tuberal hypothalamus. Scale bars: 50 µm.

We next characterized the growing tuberal/anterior hypothalamus. At 30 hpf, *shh* is detected uniformly in the hypothalamus (Fig. 3A,A′): double-fluorescence *in situ* hybridization (FISH) analysis reveals extensive co-expression with *rx3* (Fig. 3D,D′, yellow arrowheads). *rx3^+^shh^+^* cells are bound rostrally and ventrally by *rx3^+^shh^−^* cells (Fig. 3D′, red arrowheads) and caudally/dorsally by *shh^+^* cells (Fig. 3D′, green arrowhead). In the co-expressing region, *rx3* is strongest dorso-caudally (Fig. 3D′). Similar expression domains are detected at 55 hpf (Fig. 3B,E) but a novel *shh^+^rx3^−^* domain now projects in the tuberal/anterior hypothalamus (Fig. 3B,E,F,F′, white arrowheads). This domain appears to be composed of cells that have downregulated *rx3*, resulting in the characteristic zone II, but is significantly (1.5-fold) longer at 55 hpf compared with 30 hpf (Fig. 3D,E, white arrows). Analysis of sections shows that in this domain, *shh* is restricted to cells that line the AR/LR (Fig. 3F′) and shows that *shh^+^rx3^−^* cells define the AR tips (Fig. 3F′, arrowheads; Fig. S1A,A′,C, red arrowheads). Our data show that zone II is characterised by *shh*^+^ AR cells, and, together with our previous data, suggests that AR tip cells are *shh^+^rx3^−^* progenitors that derive from adjacent *rx3^+^shh^+^* progenitors.

**Fig. 4.**
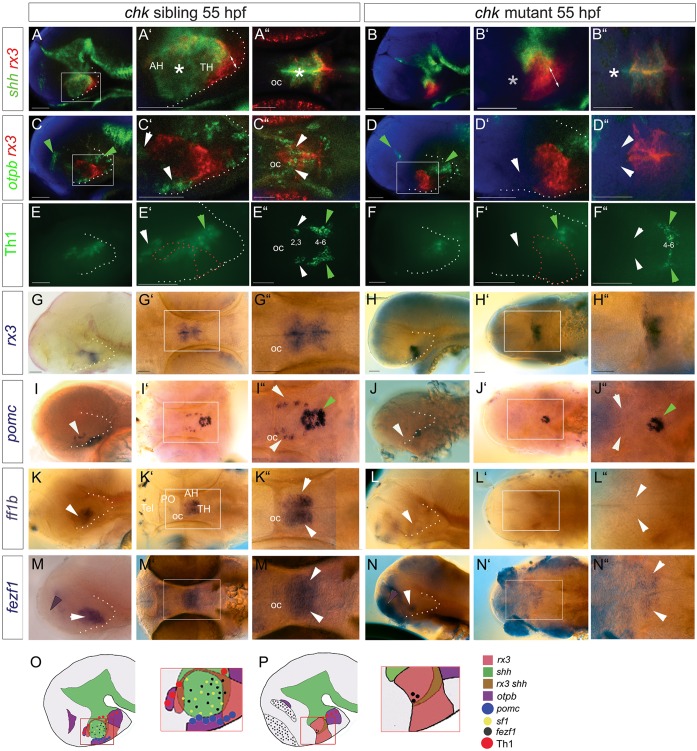
**Rx3 is required for *shh^+^* AR and anterior/tuberal differentiation.** (A-N) Side or ventral views of 55 hpf *chk* sibling or mutant embryos. Asterisks in A′,A″ show *shh^+^* AR, which is absent in the *chk* mutant (B′B″, asterisks). White arrowheads point to *otpb^+^* tuberal/anterior cells (C′,C″), Group2/3 Th1^+^ anterior cells (E′,E″), *pomc^+^* cells (I,I″), *ff1b^+^* cells (K,K″), all of which are absent in *chk* mutants (D′,D″,F′,F″,J,J″,L,L″,N,N″), and to *fezf1^+^* progenitors (M,M″), which are reduced in the *chk* mutant (N,N″). Green arrowheads point to expression domains unaffected in *chk* mutants. Purple arrowheads point to *fezf1* domain, upregulated in *chk* mutants. White and red dotted lines as in Fig. 2. (O,P) Schematics depicting expression patterns; boxed regions show areas shown in high-power views. AH, anterior hypothalamus; oc, optic commissure; PO, preoptic hypothalamus; Tel, telencephalon; TH; tuberal hypothalamus.  Scale bars: 50 µm.

In zebrafish, immature tuberal/anterior hypothalamic neurons can be characterized through expression of the transcription factor *otpb* (Eaton and Glasgow, 2007; Löhr et al., 2009; Herget et al., 2014; Manoli and Driever, 2014), the nuclear receptor *Nr5a1/Sf1* orthologue *ff1b* (Kuo et al., 2005) and the precursor polypeptide *pomc* (Liu et al., 2003; Herzog et al., 2004; Dickmeis et al., 2007; Tessmar-Raible et al., 2007; Manoli and Driever, 2014). At 30 hpf, *otpb* is detected in the posterior hypothalamus and at the DTJ (Fig. 3G,G′) but by 55 hpf additional *otpb^+^* cells are detected in the tuberal and anterior hypothalamus (Fig. 3H,H′, white arrowheads; see Eaton and Glasgow, 2007) adjacent to the *shh^+^* AR (Fig. 3I). Ventral views show that *otpb*^+^ cells in the tuberal and anterior hypothalamus are periventricular, suggesting they are immature neurons (see Fig. 4C″; Herget et al., 2014). *ff1b* expression is detected at 30 hpf (Fig. 3J,J′), and by 55 hpf is expressed broadly in the tuberal hypothalamus. Sections reveal that *ff1b* is expressed in *shh*^+^ AR cells and adjacent periventricular cells (Fig. 3K-K″). *pomc^+^* cells cannot be detected in the 30 hpf hypothalamus (Fig. 3L,L′) but by 55 hpf are detected in the tuberal hypothalamus (Fig. 3M-M″) rostral to *rx3^+^* progenitors (Fig. 3N, white arrowheads). Together, our data show that anterior elongation correlates with the development and growth of the *shh^+^rx3^−^* AR and with the differentiation of *otpb^+^*, *ff1b^+^* and *pomc^+^* cells in the tuberal/anterior hypothalamus (schematized in Fig. 3O,P).

### Rx3 is required for *shh^+^* AR and neuronal differentiation

We next addressed the requirement for Rx3 in development of the tuberal/anterior hypothalamus. Previous studies have shown that *pomc^+^* and *avp^+^* neurons are absent in embryos lacking *rx3* (Dickmeis et al., 2007; Tessmar-Raible et al., 2007) but a more extensive characterization of other progenitor/differentiating cells has not yet been performed.

Analysis of 55 hpf *chk* embryos shows that the *shh^+^* AR fails to develop in *chk* mutants (Fig. 4A-B″, white asterisks; note that posterior *shh* expression in the floor plate and basal plate appears to be unaltered). *rx3* expression itself is markedly different in *chk* mutant embryos compared with siblings: zones I-III cannot be clearly resolved (Fig. 4A′,B′,G-H″).

The failure in development of the *shh^+^* AR correlates with a failure in differentiation. Mutant embryos lack *otpb^+^* cells in both the tuberal and anterior hypothalamus [Fig. 4C-D″, white arrowheads; note that *otpb^+^* cells in the posterior hypothalamus and at the DTJ (green arrowheads) appear to be unaffected]. Previous studies suggest that the anterior *otpb^+^* progenitors give rise to Group 2/3 Tyrosine hydroxylase (Th) dopaminergic neurons (Löhr et al., 2009); in keeping with this, mutant embryos lack Group 2/3 Th1^+^ neurons (Fig. 4E-F″, white arrowheads: note Group 4-6 Th1^+^ neurons are not eliminated). *rx3* mutant embryos additionally lack *pomc^+^* cells (Fig. 4I-J″, white arrowheads) and *ff1b^+^* cells (Fig. 4K-L″, white arrowheads) in the tuberal hypothalamus [note *pomc^+^* cells in the adenohypophysis (green arrowheads) are still detected]. Finally, *fezf1*, a homeodomain (HD) gene that in mouse is regulated by *Sf1* (Kurrasch et al., 2007) and in fish regulates *otpb* (Blechman et al., 2007), is markedly reduced (Fig. 4M-N″, white arrowheads); at the same time, ectopic expression is detected in the telencephalon. *rx3* morphant embryos closely phenocopy *chk* mutants (Figs S2, S3; Fig. 6G,G′; Fig. 7N-R). Together, these analyses show that Rx3 is required for establishment of the *shh^+^rx3^−^* AR and for the differentiation of tuberal/anterior cells (Fig. 4O,P).

### Rx3 represses dorsal and ventro-tuberal progenitors

We postulated that, as in mouse (Lu et al., 2013), Rx3 may switch the identity of other progenitor domains to select posterior tuberal/anterior progenitor fates, and that the absence of Rx3 will lead to alterations in progenitor domains/increased alternative fates.

**Fig. 5.**
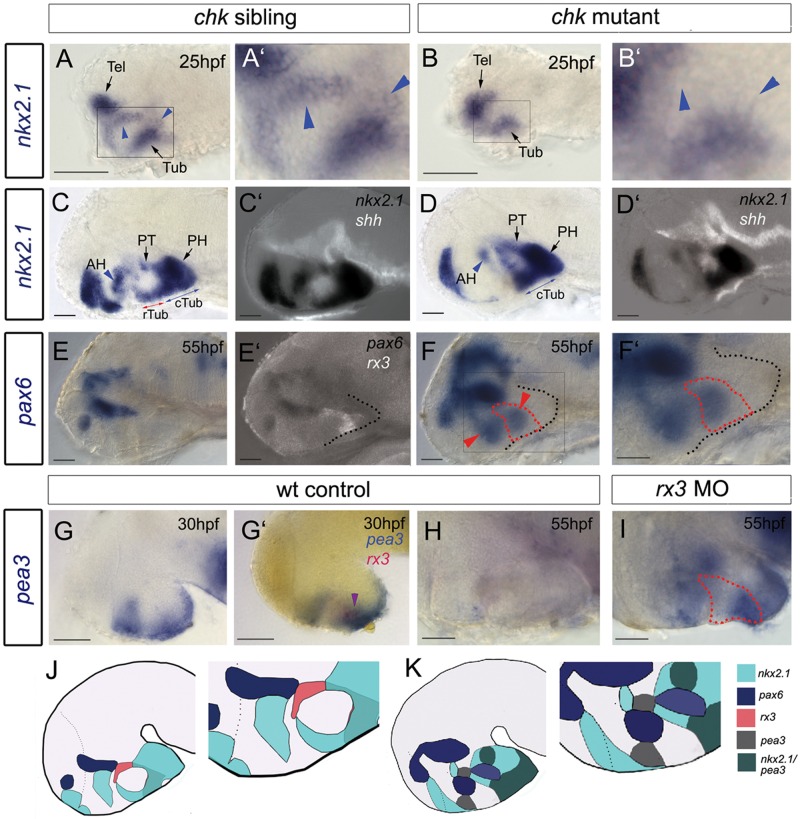
**Rx3 suppresses dorsal and ventro-tuberal progenitors.** (A-I) Side views of control embryos or embryos in which *rx3* is absent. A′,B′,F′ show high-power views of boxed regions in A,B,F. Blue arrowheads and red arrows in A-D point to *nkx2.1^+^* cells, which are absent in *chk* mutants. Blue arrows in C,D point to *nkx2.1*^+^ ventral-tuberal domain. Red arrowheads in F point to ectopic *pax6^+^* cells. Black dotted lines indicate outline of ventral hypothalamus. Red dotted lines as in Fig. 2. Purple arrowhead in G′ points to *rx3^+^pea3^+^* cells. H,I show views of isolated neuroectoderm. (J,K) Schematics of expression patterns in *chk* sibling (J) or mutant (K) 55 hpf embryos. White and red dotted lines as in Fig. 2 AH, anterior hypothalamus; PH, posterior hypothalamus; PT, posterior tuberculum; Tel, telencephalon; (c)(r)Tub, (caudal) (rostral) tuberal hypothalamus. Scale bars: 50 µm.

The transcription factor *nkx2.1* (previously known as *nkx2.1a*; Manoli and Driever, 2014), the homologue of which in mouse is required for tuberal neuronal differentiation (Correa et al., 2015; Kimura et al., 1996; Yee et al., 2009), shows subtle differences in expression in *chk* mutants at 25 hpf: two sets of *nkx2.1^+^* cells in the forming tuberal/anterior hypothalamus (Fig. 5A,A′, blue arrowheads) cannot be detected (Fig. 5B,B′). By 55 hpf, this difference is pronounced: *nkx2.1* is reduced in the anterior hypothalamus and is not detected in the rostral tuberal hypothalamus [Fig. 5C,D; position of tuberal/anterior hypothalamus confirmed through double-labelling with *shh* (Fig. 5C′,D′)]. *nkx2.1* in the caudal tuberal, posterior hypothalamus and posterior tuberculum appears to be unchanged.

Previous studies show that Nkx and Pax6 transcription factors exert cross-repressive interactions in the hypothalamus (Manoli and Driever, 2014), prompting us to examine expression of *pax6*. In control embryos, *pax6* is confined to the thalamus/dorsal hypothalamus and abuts the dorsal-most boundary of *rx3* (Fig. 5E,E′). In the absence of Rx3, *pax6* is detected ectopically in the tuberal/anterior hypothalamus within and rostral to *rx3^+^* cells (Fig. 5F, red arrowheads; Fig. S2). Thus, the absence of Rx3 leads to a ventral expansion of *pax6^+^* progenitors.

Ectopic *pax6^+^* domains do not extend throughout *rx3* zone III (Fig. 5F, red dotted outline) raising the question of whether other progenitors are also affected by loss of Rx3. The ets transcription factor *pea3* (*etv4*) is expressed in the hypothalamus at 30 hpf, and overlaps with *rx3* zone III cells (Fig. 5G,G′). *pea3* is downregulated at 55 hpf in control embryos but expression persists in *chk* mutants (Fig. 5H,I). These results suggest that Rx3 normally suppresses both dorsal *pax6^+^* and ventro-tuberal *pea3^+^* progenitors (Fig. 5J,K schematics) and predicts a widespread change in the profile of other progenitor markers in *chk* mutants. In support of this idea, *ascl1a* and *sox3* are not downregulated in zone II in *chk* mutant embryos, in contrast to their appearance in controls (Fig. S4A-H, white arrowheads).

In mouse, conditional ablation of Rx leads to a failure to select arcuate/VMN fates and, instead, additional *Dlx2^+^* DMN cells form (Lu et al., 2013). To determine whether the increase in *pea3* and *pax6* expression results in an increase in ventro-tuberal and DMN-like cells, respectively, we examined the neurohypophyseal marker *fgf3* and the DMN marker *dlx1* (*dlx1a*)*.* Both show slightly stronger expression in *chk* mutants (Fig.S4E-H). and the ventro-tuberal hypothalamus appears longer in *chk* mutants (Fig. S4A,C) suggesting that in the absence of Rx3, there is some expansion of ventral-tuberal and dorsal progenitors and their derivatives.

### Rx3 is required for progenitor survival and anisotropic growth

**Fig. 6.**
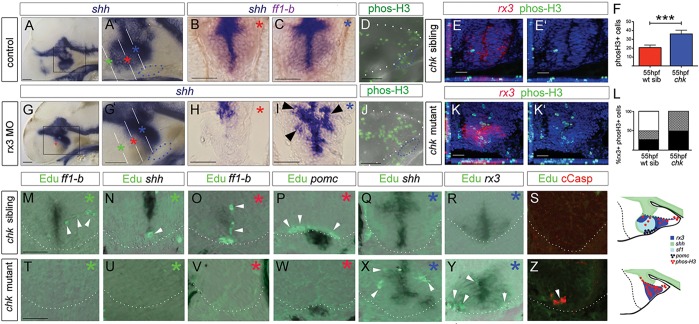
**Rx3 promotes progenitor survival and growth.** (A-C,G-I) Whole-mount side views (A,G) or sections (B,C,H,I: planes and positions indicated by coloured asterisks) through 55 hpf control or *rx3* morphant embryos. A′,G′ show high-power views of boxed regions in A,G. Arrowheads in I show disorganized *shh^+^* cells around 3V. (D,J) Whole-mount side views of phosH3 in 55 hpf control or *rx3* morphant embryo. (E,E′,K,K′) Representative sections after phosH3/*rx3* co-labelling in 55 hpf *chk* sibling or mutant embryos. E′,K′ show single-channel views. (F,L) Quantitative analyses. (F) Numbers of phosH3^+^ cells in *chk* mutant or sibling embryos (*n*=6 each). Significantly more phosH3^+^ cells are detected in mutants compared with siblings (*P*<0.001). (L) Proportion of phosH3^+^ cells that are *rx3^+^* (black), adjacent to (hatched) or distant from (white) *rx3^+^* cells in mutant versus sibling *chk* embryos. (M-R,T-Y) Representative serial sections, from rostral to caudal (coloured asterisks denote approximate position of each section, see A′,G′) of a 55 hpf *chk* sibling (M-R) or mutant (T-Y) embryo. White arrowheads point to EdU^+^ cells. (S,Z) Representative sections of 35 hpf *chk* sibling or mutant embryos. 18±2 cCasp^+^ cells detected in *chk* mutants, *n*=8 embryos. Schematics summarize expression patterns in mutant versus sibling *chk* embryos. White and blue dotted lines as in Fig. 2. Scale bars: 50 µm.

The increase in *fgf3* and *dlx1* in *chk* mutants is, however, mild, suggesting that Rx3 may play a role other than switching progenitor fates. In sectioned embryos we had noticed an unusually disorganized accumulation of *shh*^+^ cells (Fig. 6A-C,G-I) suggesting that some ectopic progenitors may accumulate in the recess walls, rather than grow and progress to normal fates.

To examine this further, we compared proliferation and fate in control and *rx3*-null embryos. In comparison to controls, *rx3*-morphant and *chk* mutant embryos showed significantly more phosH3^+^ cells in the 55 hpf embryo (Fig. 6D-F,J-K′) that, in contrast to controls, were largely *rx3^+^* or adjacent to *rx3^+^* cells (Fig. 6L). To determine more specifically the fate of proliferating progenitors, we pulsed 30 hpf fish with EdU, chased to 55 hpf and, on serial adjacent sections, analysed whether EdU^+^ cells progressed to periventricular cells in the tuberal/anterior hypothalamus, were retained as *rx3^+^* or *shh^+^* progenitors, or assumed other fates. In *chk* siblings, the majority (63%; *n*=156 cells, 6 embryos) of EdU^+^ cells were laterally oriented chains in the anterior (Fig. 6M) or tuberal (Fig. 6P) hypothalamus and were detected in or in the vicinity of *ff1b^+^* and *pomc^+^* cells (Fig. 6M,O,P). A minority (27%) were *shh^+^rx3^−^* anterior (Fig. 6N,O) or lateral (not shown) recess tip cells. No EdU^+^ *rx3^+^* cells were detected in zones I or III (Fig. 6R; data not shown). By contrast, in *chk* mutant embryos, no EdU^+^ cells were detected in the region rostral to the adenohypophysis, i.e. the region that would form part of the anterior/tuberal hypothalamus (Fig. 6T-W). The majority (76%, *n*=165 cells, 6 embryos) of EdU labelling was detected in/adjacent to *shh^+^* (Fig. 6X) and *rx3^+^* (Fig. 6Y) cells. EdU^+^ cells accumulated especially at the recess junctions and tips. No cleaved (c)Caspase^+^ cells were detected after the 25 h chase period, but after a 5 h chase, cCaspase^+^ cells, including EdU^+^cCaspase^+^ cells were detected in *chk* mutants (Fig. 6Z). No cCaspase was detected in siblings (Fig. 6S).

These findings, together with our previous observations, suggest that *rx3^+^* progenitors give rise to cells, including *shh^+^* AR tip cells, that grow anisotropically and give rise to anterior/tuberal cells. Additionally, these findings show that in the absence of Rx3 function, many progenitor cells accumulate in the recesses, where they either die, or fail to differentiate. Together, these observations point to a mechanism in which Rx3 selects tuberal/anterior progenitors and governs their survival and growth (Fig. 6 schematics).

### Shh is an ‘on-off’ switch for *rx3*

Our findings demonstrate that Rx3 is upstream of that of *shh* in the tuberal/anterior hypothalamus. However, given the crucial role of Shh in induction and early patterning of the hypothalamus (Bedont et al., 2015; Burbridge et al., 2016; Pearson and Placzek, 2013; Blaess et al., 2015), we wished to test whether at earlier stages of hypothalamic development Shh is upstream of *rx3*, a possibility suggested by the observation that at epiboly stages, *shh* is expressed on midline cells, close to the early zone of *rx3* expression (Fig. S5A,B).

**Fig. 7.**
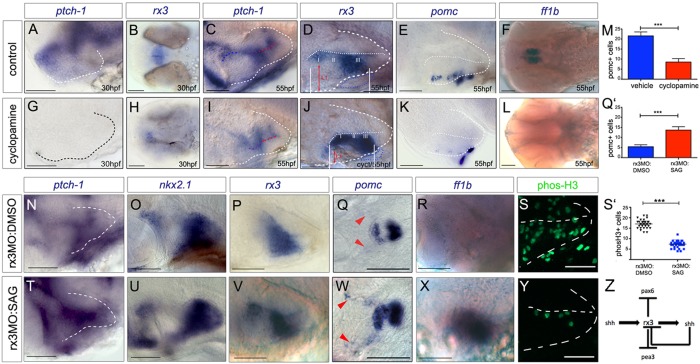
**Shh signalling functions as an *rx3* ‘on-off’ switch.** (A-L) Side or ventral views of 30 hpf and 55 hpf wild-type embryos, exposed to vehicle or cyclopamine over 10-28 hpf (A,B,G,H) or over 28-55 hpf (C-F,I-L). Red arrows and white bars in D,J show distances measured for width and length of tuberal/anterior hypothalamus. (M) Quantitative analysis: significantly fewer *pomc*^+^ cells are detected after cyclopamine exposure (***P<0.0001, *n*=30 embryos). (N-Y) Side or ventral views of 55 hpf *rx3* morphant embryos, exposed to DMSO vehicle (N-S) or SAG (T-Y) from 28 hpf. (Q′,S′) Quantitative analysis. Significantly more *pomc+* cells (Q'; ***P<0.0001, *n*=30 embryos) and significantly fewer phosH3^+^ cells (S'; ***P<0.0001, *n*=27 embryos) are detected after late SAG rescue. (Z) Model for anterior/tuberal progenitor development. Scale bars: 50 µm.

*Ptch1*, a Shh-receptor and ligand-dependent antagonist, is weakly detected in the forming tuberal/anterior hypothalamus at 30 hpf (Fig. 7A), but not detected when embryos are exposed to cyclopamine over 10-28 hpf (Fig. 7G). Similar observations were made with *ptch2* (not shown). At the same time, cyclopamine treatment results in a marked downregulation of *rx3* (Fig. 7B,H) mimicking the phenotype of *slow muscle omitted* (*smu*) mutant zebrafish that lack essential components of the Hh pathway (Fig. S5C,D). Together, these results suggest that Shh induces *rx3* in the early embryo.

By 55 hpf, strong *ptch1* expression is detected in zones I and III (Fig. 7C) with weaker expression in zone II (Fig. 7C). *ptch2* expression appears similar (not shown). To determine whether Shh influences *rx3* at this stage, we exposed embryos to cyclopamine over 28-55 hpf. This resulted in an effective inhibition of Shh signalling, as judged by *ptch1* downregulation (Fig. 7I) but led to a consistent increase in *rx3* expression (Fig. 7D,J). Increased *rx3* expression was accompanied by changes that appeared to phenocopy loss of *rx3*, notably a significant decrease in tuberal/anterior territory (Fig. 7D,J white lines and red arrows), a decrease in hypothalamic *pomc*^+^ cells (Fig. 7E,K,M), the loss of *ff1b* expression (Fig. 7F,L), a decrease in Th1^+^ Group2/3 neurons (Fig. S5E,F; note Groups 4-6 in the posterior hypothalamus are unaffected) and a failure to downregulate *sox3* in zone II (not shown). These observations suggest that Shh mediates *rx3* downregulation in zone II, and that this is essential for differentiation of tuberal/anterior hypothalamic progenitors.

This idea predicts that provision of Shh may be sufficient to rescue the phenotypic effects of *rx3* morphant embryos, once the effects of the morpholino begin to disappear. To test this, we attempted a ‘late rescue’, in which *rx3* morphant embryos were exposed to the small molecule Shh agonist SAG over 28-55 hpf. SAG was effective in restoring a normal pattern of Shh signalling in *rx3* morphant embryos, as judged by expression of *ptch1* (Fig. 7N,T). Furthermore, both the normal pattern of *nkx2.1* and the characteristic profile of *rx3* in zones I, II and III were restored (Fig. 7O,P,U,V). Both *pomc^+^* and *ff1b^+^* cells were restored in *rx3* morphant embryos in response to SAG administration (Fig. 7Q,Q′,R,W,X). Finally, cellular homeostasis was restored: the enhanced numbers of phosH3^+^ cells in *rx3* morphants were reduced to normal, wild-type levels (Fig. 7S,S′,Y). This rescue is not seen when an early SAG-treated regime is used (10-28 hpf; not shown), or in *chk* mutant embryos treated with SAG over 28-55 hpf (Fig. S6), indicating that functional Rx3 is required for the late rescue. Together, these results suggest that a Shh-*rx3* ON and Shh-*rx3* OFF feedback loop (Fig. 7Z) is essential for the development of the tuberal/anterior hypothalamus.

## DISCUSSION

Here, we show that Rx3 function is required for morphogenesis of the tuberal/anterior hypothalamus and governs three aspects of cell behaviour: it re-specifies progenitor types to tuberal/anterior identities, promotes their survival and governs their anisotropic growth/migration. Shh coordinates tuberal/anterior progenitor selection and behaviour by acting as an on-off switch for *rx3*. Thus, a Shh-Rx3-Shh feed-forward/feedback loop generates tuberal/anterior progenitors that grow to expand the surface area of the third ventricle and diversify the neuronal subtypes that differentiate around it.

### Rx3 selects tuberal/anterior hypothalamic progenitors

Our studies confirm that Rx3 function is not required for induction or initial hypothalamic patterning (Kennedy et al., 2004), but show that it is essential to elaborate patterning. Our data suggest that Rx3 autonomously selects *nkx2.1^+^* tuberal/anterior progenitors that grow anisotropically. In *chk* mutant embryos, *pax6a* expands ventrally into *rx3^+^* progenitors, a phenotype detected as early as 19 hpf (Loosli et al., 2003). The ventral expansion of *pax6a* mimics the phenotype of *nkx2.1/nkx2.4a/nkx2.4b-*null embryos (Manoli and Driever, 2014) and suggests that Rx3 re-specifies progenitors that would otherwise assume a dorsal hypothalamic or pre-thalamic identity.

At the same time, Rx3 represses *pea3*. In wild-type animals, *pea3* overlaps with the ventral-most domain of *rx3* expression at 30 hpf, but is downregulated by 55 hpf. In *chk* mutant fish, *pea3* expression persists. Although we have not performed double FISH with *pea3* and *pax6* in *chk* mutant fish, their expression patterns appear to be complementary. This suggests that Rx3 operates as a switch in at least two separate progenitor populations and provides a prosaic interpretation for the existence of two domains: the dorsal *rx3^+^shh^+^* and ventral *rx3^+^shh^−^* domains.

Our studies reveal that Rx3 promotes alternative fates in progenitor cells. Its loss leads to one of three outcomes: to undergo apoptosis or to be retained as a proliferating cell held in the wall of the ventricle (novel outcomes), or to initiate alternative adjacent differentiation programmes – after pulse-chase, some EdU^+^ cells are detected in periventricular regions in *chk* mutants where it is likely that they contribute to *nkx2.1^+^pea3^+^* progenitors and hence *fgf-*expressing neurohypophysis ventrally, and to *dlx1^+^* cells dorsally. *dlx1^+^* cells are likely to be immature DMN-like neurons and notably, *somatostatin^+^* neurons persist in *chk* mutants (Dickmeis et al., 2007). Together, our studies suggest that Rx3 selects tuberal/anterior neuronal progenitors and limits both ventro-tuberal neurohypophyseal and DMN-like progenitors.

In addition to promoting cell survival, Rx3 regulates cellular homeostasis in the tuberal/anterior hypothalamus, orchestrating a balance of proliferation and differentiation. We surmise that the increased proliferation seen in the absence of Rx3 reflects changes in Wnt or Fibroblast growth factor signalling, both of which are upregulated in *chk* mutants (Stigloher et al., 2006; Yin et al., 2014; this study). *fgf3*, in particular, normally abuts neuroepithelial-like *rx3^+^shh^−^* cells in both zones I and III and is upregulated in *rx3* mutants. Potentially, the driving force for proliferation resides in *rx3^+^shh^−^* cells in zones I and III that progress to *rx3^+^shh^+^* cells in zone II.

Previous reports have shown that Rx3 is required for retinal fate selection and that telencephalic fates are expanded in its absence (Bielen and Houart, 2012; Cavodeassi et al., 2013). Our studies likewise show changes in the telencephalon/eye territory: *fezf1* is upregulated in *rx3* mutants, and both *shh* and *nkx2.1* in the telencephalon/tuberal/anterior area are greatly reduced. Together, these studies suggest that Rx3 selects fate in cells of distinct origins: anterior telencephalic and posterior diencephalic. Importantly, not all hypothalamic cells alter their identity in the absence of Rx3: the posterior hypothalamus expresses *nkx2.1*, *shh* and *otpb* as normal, the rostral-most hypothalamus expresses *otpb* and *nkx2.1* and the tuberal hypothalamus expresses *nkx2.1*, *pea3* and *fgf3*, emphasising the fact that Rx3 elaborates, rather than initiates, hypothalamic patterning.

### Shh is an on-off switch for *rx3*

Our study shows that Shh is required for both the induction of *rx3* and the progression of *rx3^+^* to *rx3^−^shh^+^* progenitors and demonstrates that both steps are required for tuberal/anterior hypothalamic neurogenesis. Downregulation of Shh signalling over 10-30 hpf leads to an almost complete loss of *rx3* expression. By contrast, downregulation over 30-55 hpf leads to sustained *rx3* in zone II and a phenotype that is highly similar to that of *chk* mutants: *sox3* is not downregulated in zone II, the *shh^+^rx3^−^* AR does not form, the tuberal/anterior hypothalamus is short and its resident neurons do not differentiate. Importantly, the Shh agonist SAG can restore normal patterns of proliferating progenitors and neuronal differentiation in late *rx3* morphants. The most likely interpretation of these findings is that Shh-mediated *rx3* upregulation is required to select tuberal/anterior progenitors but that Shh-mediated *rx3* inhibition is required for these to realise their differentiation programme(s). Future studies are needed to establish whether the downregulation of *sox3*, *nkx2.1*, *ascl1* and *ptch1* that we observe in wild-type but not *chk* mutant fish are similarly required for progression of tuberal/anterior progenitors. We predict that the downregulation of *ptch1*, in particular, supports Shh active signalling from zone II cells and contributes to development of the *shh*^+^ AR. The intricate regulation of induction and cessation of Shh signalling in sets of neighbouring cells is emerging as a common theme within the CNS (Briscoe and Therond, 2013) and provides the opportunity to drive expansion of territories and build increasingly complex arrays of neurons.

In summary, our studies suggest that Shh plays a dual role in *rx3* regulation, inducing, then repressing it, and are consistent with a model in which Shh deriving from AR cells, feeds back to *rx3^+^* progenitors to promote their further differentiation.

### Origins of hypothalamic neurons

Our studies show that the zebrafish tuberal hypothalamus includes regions analogous to the mouse Arc and VMN. Our EdU pulse-labelling studies suggest that *shh^+^* AR cells and differentiating *ff1b^+^* and *pomc^+^* neurons derive from *rx3^+^* cells. After a 25 h chase, we detect strings of EdU^+^ cells, presumably of clonal origin, extending medio-laterally from the *shh^+^* AR tips to *pomc^+^* and *ff1b^+^* regions, favouring the idea that forming neurons derive from *rx3^+^shh^+^* progenitors via *rx3^−^shh^+^* progenitors. In mouse, *Rax^+^* cells give rise to *Pomc^+^* and *Sf1^+^* neurons (Liu et al., 2013). Other mouse studies show that *Shh^+^* hypothalamic cells give rise to tuberal neurons (Alvarez-Bolado et al., 2012), and that *Shh* ablation in hypothalamic cells leads to the loss of *Pomc* and *Sf1* (Shimogori et al., 2010) and a reduction in hypothalamic territory (Alvarez-Bolado et al., 2012; Zhao et al., 2012). These studies, together with observations that loss of Nkx2.1 results in loss of tuberal hypothalamic neurons (Correa et al., 2015; Kimura et al., 1996; Yee et al., 2009), disruptions to the infundibulum and a reduction in the size of the third ventricle (Kimura et al., 1996) suggests a conserved differentiation route of *pomc* and *ff1b/sf1* immature neurons and the tuberal hypothalamus from zebrafish to mouse.

In zone I, *rx3* is expressed in the anterior hypothalamus, in a region that may be equivalent to the anterior-dorsal domain reported in mouse (Shimogori et al., 2010). Our work, together with previous studies, suggests that here, Rx3 plays a role in a conserved differentiation pathway for *avp^+^* and Group2/3 Th1^+^ neurons. *avp^+^* and Group 2/3 Th1^+^ neurons localize within a discrete subregion of hypothalamic *otp* expression (Löhr et al., 2009; Herget et al., 2014; Herget and Ryu, 2015) and in fish, as in mouse, *otp* genes are required for the differentiation of neurons that express *avp* and Th (Acampora et al., 1999; Löhr et al., 2009; Fernandes et al., 2013). *avp^+^* neurons fail to differentiate in the absence of *rx3* (Tessmar-Raible et al., 2007) and we now show a specific loss of an *otpb^+^* subset and Group 2/3 Th1^+^ neurons. This suggests that Rx3 governs a subset of *otpb^+^* progenitors in the anterior hypothalamus that will give rise to *avp^+^* and Group 2/3 Th1^+^ neurons. We have not yet investigated whether this *otpb^+^* progenitor subset are dependent on Shh. However, in mouse, conditional deletion of hypothalamic Shh leads to a reduction in *Otp* expression and *Avp^+^* neurons (Szabo et al., 2009) as well as a loss of *Sim1* in the PVN (Shimogori et al., 2010), suggesting that the Shh-Rx3-Shh pathway that governs *pomc*^+^ and *ff1b*^+^ cell fates may likewise govern *avp^+^* and Group 2/3 Th1^+^ fates. A previous study has highlighted Sim1 and Otp as core components of a conserved transcriptional network that specifies neuroendocrine as well as A11-related hypothalamic dopaminergic neurons (Löhr et al., 2009), suggesting that Rx3 may be intimately linked to this pathway. Notably, because other NPO neurons, including *oxytocin*^+^ (previously known as *isotocin*) neurons are not affected by loss of Rx3, our data suggest that neurons that make up the NPO derive from discrete lineages. Our work adds to a growing body of evidence that directed cell migrations play a pivotal role in ventral forebrain/hypothalamic morphogenesis (Varga et al., 1999; Cavodeassi et al., 2013 and see Pearson and Placzek, 2013). We do not know the mechanisms that operate downstream of Rx3 to govern appropriate migration, but Eph/Ephrin signalling, expression of Fgf and Netrin, all of which govern cell adhesion and migration of neural cells, are disrupted in *chk* mutant embryos (Cavodeassi et al., 2013; Yin et al., 2014; this study) and could contribute.

In conclusion, our study suggests a mechanism by which Shh elaborates patterning in the hypothalamus. Previous reports suggest that Shh patterns the early hypothalamus in many vertebrates, establishing early progenitor domains (reviewed by Pearson and Placzek, 2013; Blaess et al., 2015). Our study shows that in zebrafish, Shh elaborates early patterning by switching progenitor domain identity, and promoting the survival and anisotropic growth of the new progenitor cells. Recent studies in the developing spinal cord show that the coordination of growth and specification can elaborate patterning in an expanding tissue, if molecularly distinct neural progenitor domains undergo differential rates of differentiation (Kicheva et al., 2014), raising the possibility that Shh may govern differentiation rates in the tuberal/anterior hypothalamus. Studies in mice that reveal similarities in the phenotypes of embryos in which Shh or Rax are conditionally ablated raise the possibility that features of the mechanism that we describe here may be conserved in other vertebrates.

Finally, the Shh-Rx3-Shh loop that we describe provides a means to maintain a dynamic balance between proliferating and differentiating cells. Studies in mice show that at least a subset of *Rax^+^* cells persist into adulthood as stem cells (Miranda-Angulo et al., 2014) that can direct hypothalamic neurogenesis even in postnatal life. The exquisite regulation of Shh, Fgf and Wnt signalling, via Rx3, is likely to hold the key to a better understanding of hypothalamic neurogenesis throughout life and support a better understanding of complex human pathological conditions and dysfunctional behaviours that are underlain by tuberal/anterior hypothalamic cells and circuits.

## MATERIALS AND METHODS

### Animals

Zebrafish were staged according to Kimmel et al. (1995). *chk^w29^* fish were kindly provided by Dr Breandan Kennedy (University College Dublin, Ireland).

### Nomenclature

We use the terms preoptic, anterior, tuberal and posterior to define the rostro-caudal domains of the hypothalamus. The region we define as anterior may overlap with the region that is conventionally termed the NPO (see Discussion).

### *In situ* hybridization

Single and double *in situ* hybridization methods were adapted from Thisse and Thisse (2008) and Lauter et al. (2011) (details in supplementary Materials and Methods). Embryos were post-fixed in 4% paraformaldehyde and visualized by Olympus Nomarski or confocal microscopy. For cryostat sectioning, embryos were re-fixed and equilibrated in 30% sucrose, and 15-µm-thick serial adjacent sections cut. *n*=10-40 embryos for whole mounts; *n*=4-6 embryos for sections.

### EdU analysis

Embryos were pulsed with 300 μM EdU for 1 h on ice, chased for 1, 5 or 25 h, then processed for cryostat sectioning and double EdU/*in situ* hybridization analysis (details in supplementary Materials and Methods) using the Click-iT EdU Alexa Fluor 488 Imaging Kit (Fisher Scientific).

### Immunohistochemistry

Anti-phosH3 (06-570, Millipore), anti-cleaved Caspase (9661, Cell Signaling Technology) and anti-Th1 (22941, Immunostar) were used at 1:1000. Fixed embryos or sections were processed according to Liu et al. (2013) and mounted in VectaShield.

### Length measurements

Length was determined through measurements of images, where *in situ* patterns could be detected relative to morphological landmarks (diencephalic-telencephalic junction, optic commissure, lateral ventricle, posterior hypothalamus and adenohypophysis). For each experiment, length was normalized to the average length of age-matched sibling controls.

### Cell quantification

phosH3^+^ and EdU^+^ cell numbers were obtained through counts in serial adjacent sections through individual hypothalami using *in situ* patterns against morphological landmarks (above) to determine relative position. For *chk* mutants, section position was determined relative to unaffected posterior hypothalamus.

### Image acquisition

Differential interference contrast or fluorescence images were acquired using Olympus BX60 Zeiss Confocal LSM510 Meta or Olympus Confocal microscopes. Data was processed with Adobe Photoshop CS3/Adobe Illustrator CS.

### Statistical analysis

Statistical analyses were performed using Prism 5. Each data value sampled was tested for Gaussian distribution prior to unpaired *t*-test by performing baseline subtraction of the two datasets and analysed using the D'Agostino–Pearson omnibus normality test.

### Cyclopamine treatment

Cyclopamine (in ethanol) was used at 50 µM, optimised on the basis of *ptch1* downregulation (20, 50, 100, 120 μM tested). Cyclopamine or ethanol were added to dechorionated embryos, which were kept in the dark.

### SAG treatment

SAG (Millipore-EMD chemicals) in DMSO was used at 10 µM, optimised on basis of *ptch1* upregulation (2, 5, 8, 10 μM tested). SAG or DMSO was added to de-chorionated embryos in E3 medium, and embryos were kept in the dark.

### Morpholino

Morpholinos [0.25 mM *rx3* ATG (targets TSS) and 0.15 mM *rx3* E212 (targets splice site)] (GeneTools, LLC) (Tessmar-Raible et al., 2007) were injected into one-cell embryos and morphants were selected on the basis of absent eyes.

### Note added in proof

Since acceptance of this paper, a paper by Orquera et al. (2016) suggests that in mouse, Rax governs similar mechanisms to those we describe here.
